# DARA: a web server for rapid search of structural neighbours using solution small angle X-ray scattering data

**DOI:** 10.1093/bioinformatics/btv611

**Published:** 2015-10-25

**Authors:** Alexey G. Kikhney, Alejandro Panjkovich, Anna V. Sokolova, Dmitri I. Svergun

**Affiliations:** ^1^European Molecular Biology Laboratory, Hamburg Outstation, EMBL c/o DESY, 22607 Hamburg, Germany and; ^2^Bragg Institute, ANSTO, Lucas Heights, 2234 NSW Australia

## Abstract

**Motivation:** Small angle X-ray scattering (SAXS) is an established method for studying biological macromolecules in solution, whereby the experimental scattering patterns relate to the quaternary and tertiary structure of the macromolecule. Here we present DARA, a web-server, that queries over 150 000 scattering profiles pre-computed from the high resolution models of macromolecules and biological assemblies in the Protein Data Bank, to rapidly find nearest neighbours of a given experimental or theoretical SAXS pattern. Identification of the best scattering equivalents provides a straightforward and automated way of structural assessment of macromolecules based on a SAXS profile. DARA results are useful e.g. for fold recognition and finding of biologically active oligomers.

**Availability and implementation:** http://dara.embl-hamburg.de/

**Contact:** svergun@embl-hamburg.de

## 1 Introduction

In a small angle X-ray scattering (SAXS) experiment a homogeneous solution of proteins, nucleic acids or their complexes is illuminated by a monochromatic X-ray beam. The isotropic scattering intensity *I*(*s*) is measured as a function of momentum transfer *s* = 4π*sin*(θ)/λ, 2θ being the scattering angle and λ the X-ray wavelength. For monodisperse solutions, the signal after background subtraction is proportional to the scattering of a single particle averaged over all orientations.

The scattering pattern contains information about the macromolecular structure at a low resolution (1–2 nm). Without a priori information, *ab initio* particle shape can be reconstructed. If an atomic model is available, its theoretical scattering can be computed and compared to the experimental data (Svergun *et al.*, 1995). Comparison of the experimental scattering to a manifold of known scattering patterns is a useful alternative to shape determination. If one or several patterns agree with the experimental data the corresponding models may provide insights about the quaternary and sometimes also tertiary structure.

Here, we present DARA, a web-server that rapidly compares over 150 000 scattering profiles pre-computed from models in the Protein Data Bank (PDB) (Berman *et al.*, 2007) with a given SAXS pattern. The current implementation of DARA represents an overhaul of the database designed for proteins over twelve years ago (Sokolova *et al.*, 2003). The implementation features a new search algorithm combining principal component analysis and *k*-d trees for almost instantaneous (within a few seconds) identification of the scattering neighbours, includes nucleic acids and complexes in the search space, and provides an enhanced presentation of the results.

## 2 Methods

### 2.1 Preparing the simulated SAXS data

In DARA, all PDB entries are grouped into three categories: proteins, nucleic acids and protein:nucleic acid complexes. For each entry all biological assemblies were retrieved; from nuclear magnetic resonance entries only the first model was taken into account. Each model is examined for connectivity, and models with domains separated by more than 7 Å are excluded. PDB is regularly checked for new and obsolete models.

Theoretical scattering curves were computed with CRYSOL 2.8 from *s* = 0 to *s* = 1.0 Å^−^^1^ on a grid of 256 points using 50 orders of spherical harmonics. This was sufficient to cover models with maximum intra-particle distance *D*_max_ up to 800 Å; larger models (less than 0.03% of all PDB) were excluded.

For each model CRYSOL calculated its *D*_max_, radius of gyration (*R*_g_), molecular weight (MW) and excluded volume of the hydrated particle (*V*). For proteins, secondary structure content was computed as the percentage of alpha helices and beta sheets. The resulting curves were normalized to *I*(0) = 1 and the intensities were multiplied by *s*^2^, i.e. internally the data were treated as *s*^2^*I*(*s*) versus *s* (so-called Kratky plot representation).

PDB contains many structurally similar models (e.g. same proteins at different resolution or close homologs) yielding very similar SAXS profiles. To reduce the redundancy the curves were clustered into groups that have mutual Euclidean distance between the curves below a threshold chosen to have the number of clusters roughly half of the total number of all models. Each cluster is represented by the model with the best ‘overall quality’ as provided by the PDB. Further processing described below was applied only to the curves from the representative models.

### 2.2 Principal component analysis and *k*-d trees

We used principal component analysis (PCA) to reduce the dimensionality of the space of the computed SAXS data. Each scattering vector was treated as a single point in a 255-dimensional space, where the first dimension is the lowest nonzero angle in the momentum transfer vector, the second dimension is the second value and so on. PCA was carried out by the means of R (R Development Core Team, 2008) *prcomp* function which uses singular value decomposition to generate a new set of axes (principal components, PCs) for the scattering vectors maximizing the spread of the data. The first PC has the largest possible variance, each following PC is orthogonal to the preceding one and accounts for the largest possible variability in the data. To effectively compress the data we used up to 15 principal components, which allowed for a reasonable reconstruction of complex scattering vectors.

We further built *k*-d trees using the compressed data. The *k*-d tree is a binary tree in which each node is a *k*-dimensional point. Each non-leaf node in the tree splits the search space in two regions containing half of the points of the parent region. The structure of the tree allows for a rapid search of the nearest neighbours via traversal of the tree from the root to a leaf by evaluating the query point at each split.

### 2.3 Input data

DARA accepts the scattering patterns in various formats.

1. Experimental data processed by GNOM (Svergun, 1992; the only input format for the previous version of DARA). The GNOM output file contains the original experimental data with errors and the regularized data extrapolated to *I*(0). The latter data are read and used by DARA and this allows one to make the search significantly more stable to experimental errors.
2. Experimental data in a three-column text format containing: momentum transfer s (first column), experimental intensity *I*(*s*) (second column) and the associated errors (third column). In this case an automatic GNOM run is performed by DARA in order to obtain the regularized curve extrapolated to *I*(0).
3. Simulated data, where the first column contains the momentum transfer s (starting from *s* = 0), and the second column contains *I*(*s*). It is also possible to submit a model in the PDB format (up to 1 MB), in this case the scattering pattern is calculated on the fly using CRYSOL. For models exceeding 1 MB we recommend to run CRYSOL locally with the settings mentioned in Section 2.1 and submit the predicted intensity.

### 2.4 DARA output

**Fig. 1.**
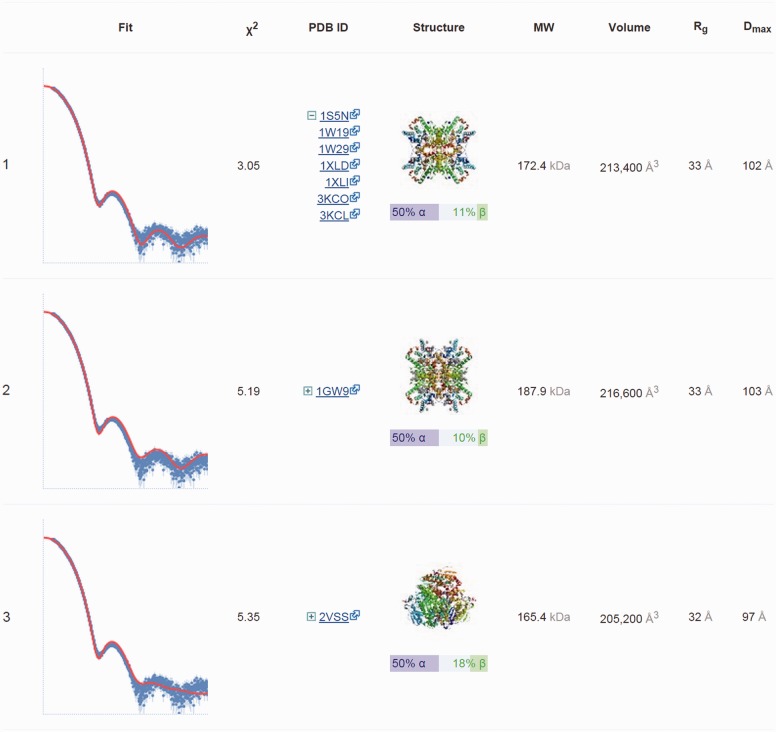
Top three nearest neighbours for experimental SAXS data collected from glucose isomerase in a phosphate buffer

DARA queries the *k*-d tree for 100 nearest neighbours, and for each curve its discrepancy *χ*^2^ with the experimental data (or its R-factor for simulated input data) is calculated, the neighbours are ranked such that the best fitting curve is shown first. For each neighbour the fit to the input data is plotted, the PDB ID and the thumbnail image of the corresponding model is shown, MW, *V*, *R*_g_ and *D*_max_ are retrieved from the database (Fig. 1). If a neighbour represents a cluster with multiple similar curves the PDB IDs corresponding to the other cluster members can be expanded as shown for the first entry on Figure 1. The list of neighbours can be downloaded.

## 3 Conclusion

DARA queries the catalogue of theoretical scattering patterns, representing the complete structural repertoire available at the PDB, to rapidly find nearest neighbours of a given (experimental or computed) SAXS profile. Given the inherent ambiguity of SAXS data interpretation in terms of three-dimensional models the neighbours will always contain false positives illustrating possible ambiguity of the SAXS-based interpretation. Still, the visualized space of conformations with similar scattering provide valuable insight on the quaternary and sometimes tertiary structure of the particle. Given the rapidly growing applications of biological solution SAXS we expect the new version of DARA to be useful for a broad community of structural biologists.
